# Fabrication of 3D Fingerprint Phantoms via Unconventional Polycarbonate Molding

**DOI:** 10.1038/s41598-018-27885-1

**Published:** 2018-06-25

**Authors:** Clayton W. Schultz, Jessica X. H. Wong, Hua-Zhong Yu

**Affiliations:** Department of Chemistry, Simon Fraser University, Burnaby, British Columbia V5A 1S6 Canada

## Abstract

Fingerprint biometrics is a valuable and convenient security tool; every fingerprint is highly detailed and unique, we always have them on “hand”. Herein we describe a novel bench-top method of making 3D fingerprint replicas (namely, fingerprint phantoms) by exploring a unique microfabrication approach using conventional polymeric materials, to aid the development of reliable and accurate fingerprint biometrics. By pressing an impression of human fingerprints onto solvent-softened plastic plates (e.g., polycarbonate chips), followed by casting with polydimethylsiloxane (PDMS, a popular elastomer), we can produce a flexible, nanoscale detailed, 3D reproduction of the fingerprint (“phantom”). By testing with standard optical fingerprint scanners, we have shown that all three levels of fingerprint details can be precisely recorded and match well with the original fingerprint. Superior to artificial fingerprint patterns, these phantoms have the exact 3D features of fingerprints and introduce no variability compared to human sampling, which make them perfect targets for standardizing fingerprint scanners and for biometric applications. We envision that the microcontact replication protocol via unconventional PC molding promises a practical, bench-top, instrumentation-free method to mass reproduce many other micro/nanostructures with high fidelity.

## Introduction

The pattern of friction ridges on our fingertips form unique patterns known as fingerprints that are popularly adapted for personal identification. Forensic science relies heavily on fingerprints collected at crime scenes as evidence; fingerprint scanning systems at borders, corporate buildings, and in our mobile devices (smartphones, pads, and laptops), keep our personal identity and data safe. In all situations, fingerprint identification relies on the collection of fingerprint data for comparison and matching. Historically fingerprints were recorded by smearing ink on the fingertip and pressing it onto paper to form physical fingerprint impressions^1^. Today fingerprints are often recorded for subsequent matching with digital fingerprint scanners either standalone or as part of a mobile device. For perspective, telecommunication experts predict that 40% of all smartphones worldwide will incorporate a fingerprint scanner, a significant increase from 30% in 2016^2^. Fingerprint scanners will be ubiquitous by 2020 as most new phones (regardless of price range) include them as default^2^. Their recording mechanisms are varied; optical scanners observe lighting differences absorbed/reflected by the ridges and valleys; capacitive scanners utilize an array of micro-capacitors to resolve capacitance difference between ridges and air; ultrasounic scanners record ridge location by detecting the echo of projected acoustic pulses^1^.

Fingerprint scanners are supposed to be robust to accommodate a wide range of user conditions, and accurate to ensure correct fingerprint matching, which are in fact facing challenges of spoofing and attacking with “synthetic” fingerprints^3^. Fingerprint scanners/readers are typically first evaluated with sine wave and ronchi grating targets, which have defined feature size, relief, and grey levels^4^. By imaging targets the scanner’s resolution can be determined, its sensitivity adjusted, and operating parameters calibrated. Subsequently, actual fingerprints are sampled for quality and matching analysis with these scanners. Sampling people is costly, time consuming, and has many sources of uncontrollable errors (pressure, finger condition, sweat level, and fingerprint type) from user input. To reduce development costs manufacturers look for alternatives to testing real people, creating a demand for “phantoms” (*vide infra*) that bear the same structural and physical characteristics of human fingerprints.

An imaging phantom (or just “phantom”) is a specially designed object that mimics the properties of tissues/organs to test biomedical diagnostic devices (MRI, CT, and ultrasound machine as examples) for accuracy and resolution calibration^5–7^. The physical properties and dimensions of phantoms are accurately defined to facilitate more precise calibrations. Many types of phantoms with a large range of complexity exist today, from simple blocks of gelatinous water of certain densities to full body phantoms containing a bone-analog skeletal system, fake organs, and tissue regions mimicking muscle, skin, and fatty tissue^5,6^. The development of patterned phantoms to test fingerprint scanners has been in demand due to the exponential increase of adapting fingerprint biometric systems for both stationary and mobile electronics as mentioned above^1,2,6^. Non-permanent gelatin phantoms can be readily constructed by molding impressions of fingers into crafting plastics (Utile Plast^TM^, Freeplastic^TM^) and silicone rubbers. Gelatin phantoms approximate finger ridge relief and electrical resistance of human tissue well; however these gelatin phantoms dry out and distort quickly, for which they are not suitable for practical calibration applitions^8,9^. The state-of-the-art approach to fabricate fingerprint phantoms is the adaptation of 3D printing technology and the generation of a 3D image from a 2D fingerprint scan (simulating the ridges)^10^. Another notable progress in this field is the development of polydimethylsiloxane (PDMS) phantoms molded from a simulated ridge pattern etched in silicon, which was developed earlier by Lu *et al*. to test ultrasounic fingerprint scanners^11^.

In retrospect, lithographically created micro/nanostructured patterns can be copied by microcontact molding^12–15^, for which either polymer substrates are thermally softened then molded under high pressure^16,17^, or liquid pre-polymers are molded first then cured with UV light^18,19^. Polycarbonate (PC) has been commonly molded to mass-produce optical discs; with properly controlled temperature and pressure sub-50 nm structures can be thermally molded with PC^20,21^. As an instrumentation-free alternative, solvent-assisted molding of various polymeric materials (particularly polyurethane^22^, polystyrene^22,23^, and polymethylmethacrylate^24^) has been explored in the past two decades, for which sub-100 nm resolutions have been achieved. The fact that PC recrystallizes into μm-size aggregates (namely, spherulites) upon treatment with solvents^25,26^, have inhibited its appeal as a suitable substrate material for solvent-assisted microcontact molding.

Herein, we report a bench-top technique to construct 3D fingerprint phantoms, for which we “reinvent” PC as an ideal candidate for solvent-assisted microcontact molding. Fingerprint impressions are first molded into a solvent-softened PC substrate (“the mold”), which serves as an enduring template (the “mold”) to cast 3D fingerprint phantoms with PDMS, the most popularly used elastomer for micro/nanofabrication. These permanent 3D replicas are derived from real fingerprint impressions with nanoscale features precisely reproduced.

## Results and Discussion

### From 3D PC Mold to PDMS Fingerprint Phantom

**Figure 1:**
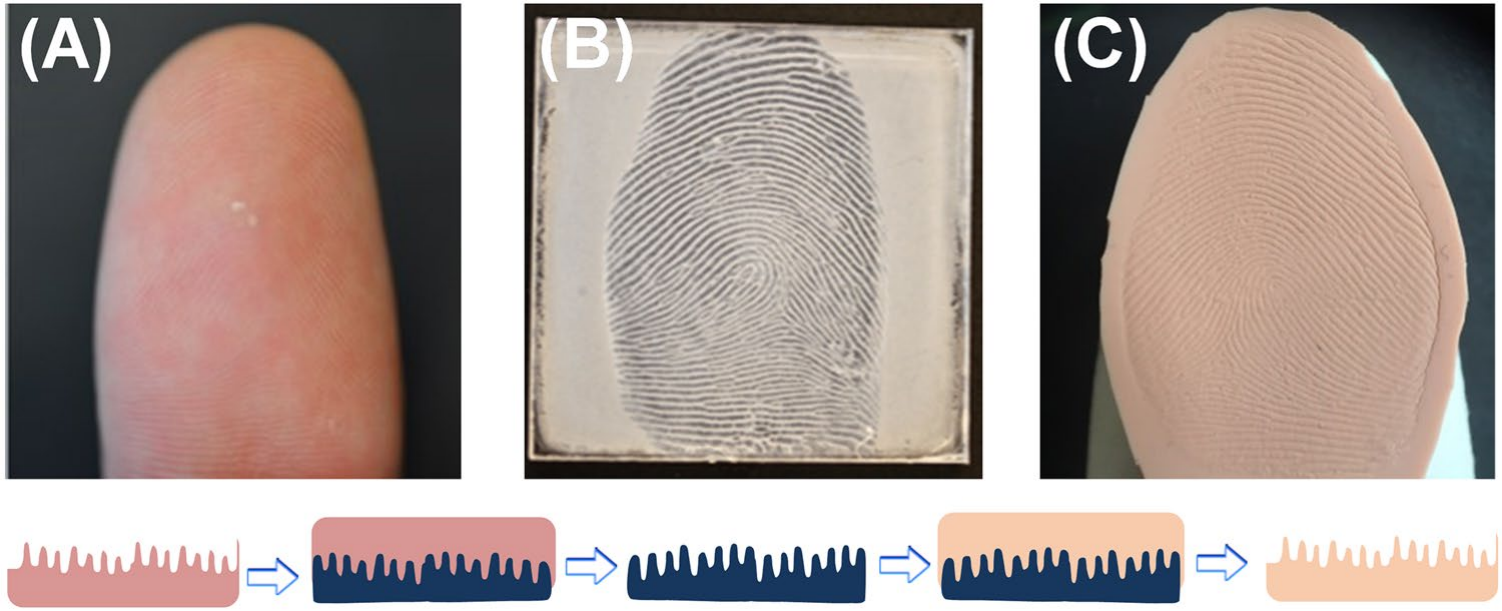
Fabrication of 3D fingerprint phantoms via solvent-assisted polycarbonate (PC) molding. (**A**) Finger to be impressed on PC. (**B**) Fingerprint impression molded on a PC plate serving as the 3D template (mold). (**C**) PDMS fingerprint phantom casted from the PC mold. The bottom insets show how fingerprint features were “copied” to PC and subsequently replicated to a PDMS phantom (color code: pink-finger, dark blue-PC mold, and beige-PDMS phantom).

The proedure to make fingerprint phantoms is not complicated, 3D physical “negatives” were first constructed on polycarbonate (PC) plates, followed by casting PDMS phantoms thereof (Fig. 1). To begin with, a thin film of acetone was sprayed onto the PC plate, which penetrates the polymer network at the PC surface causing it to swell and soften^25,26^. Upon pressing with a finger, swollen PC chains are rearranged between fingerprint ridges and into pores to form a highly detailed PC “negative” of finger ridge relief, i.e., a reusable 3D fingerprint mold (Fig. 1B). Mild skin dryness can be felt upon exposure to acetone during fingerprint molding, however, the procedure is safe because acetone does not penetrate the lipid layer to enter the bloodstream (to damage the skin)^27^. Next step is the microcontact casting using PDMS to produce the 3D fingerprint phantom (Fig. 1C). Microcontact molding was originally invented to copy defined micro/nano-patterns from silicon masters^12–15^; here we explored its unique application to replicate fingerprints via the creation of a highly-detailed 3D plastic mold at first.

A few precautions should be taken when a fingerprint phantom is fabricated. The finger was rolled from one edge of the fingernail to the other to copy the entire ridge pattern. Demolding of the fingerprint phantom from the PC mold must be done carefully; after fingerprint molding the PC surface is no longer smooth, the PDMS phantom conforming to the roughness creates a large degree of adhesion between the surfaces. On account of the flexibility of PDMS, the flat phantom can then be attached to a glove tip or wrapped around a finger before testing, which improves the usability (Fig. 1C). These fingerprint phantoms were constructed in 2–3 hours without using any sophisticated instrumentation; the materials are inexpensive PC plates and standard PDMS kits. In comparison, the wearable phantoms fabricated by Engelsma *et al*. explored a special computer algorithm to map and convert a 2D fingerprint image to a 3D molds^10^, which were focused on high resolution 3D printing and imaging processing technology. The PDMS phantoms created by Lu *et al*. were from designed silicon masters by adapting advanced lithography techniques in a clean room^11^.

**Figure 2:**
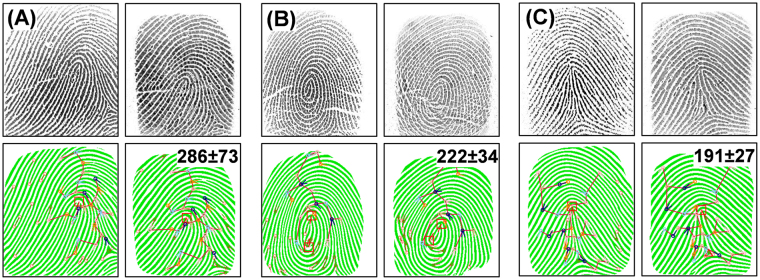
Matching of 3D PDMS phantoms with original fingerprints. (**A**) Fingerprint with a loop core. (**B**) Fingerprint with a whorl core. (**C**) Fingerprint with a tented arch core. Top row of each section: optical images of actual finger (left) and corresponding phantom (right) output from the same fingerprint scanner. Bottom row: binarized representation of fingerprint pattern with results of minutiae identification and matching algorithm overlaid. Similarity scores between original and phantom are displayed on the binarized phantom images (top left corner).

The quality of the PDMS phantoms was first examined for their fidelity with the original fingerprints they were replicated from. Fig. 2 depicts the matching of three representative fingerprints with corresponding phantoms; both images in each set were collected with the same digital fingerprint scanner (Secugen Hamster Plus). The fingerprints in (A), (B), and (C) belong to three most popular fingerprint classes (based on the core), *i.e*., a loop (65% of the population’s fingerprints), a whorl (30%), and an arch (5%) respectively^1^. Appraisal between the optical images of original fingerprint (left) and phantom fingerprint (right) illustrates the exceptional quality of the PDMS fingerprint phantoms; the phantom images are practically indistinguishable from the original fingerprints as the position and size of ridges match perfectly. The dimensions of phantom ridges are clearly defined, subtleties in ridge width and height are recorded by the scanner allowing details such as sweat pores and ridge contours to stand out. Artificially designed finger ridges, such as those produced by 3D printing of fingerprint phantoms, do not include variation in ridge width and depth that naturally occur^10^. Such distinct features will aid in optimizing scanners for imaging real fingerprints. In Fig. 2, below each fingerprint image is a binary representation; anywhere that the fingerprint matching software (Verifinger SDK) detects a ridge is displayed as solid color on a pure white background. These binarized representations allow the software to match fingerprints readily based on the location of minutiae (unique fingerprint ridge arrangements). From a research perspective it shows us where the scanner identifies ridges. Like real fingerprints, phantom fingerprints can be accurately converted to a binarized image; moreover, the locations of binarized ridges are identical to source ridges indicating that real and phantom ridges are interpreted similarly by the scanner.

Several fingerprint matching algorithms are currently available, which all operate on the same principle of comparing minutiae^1^. Particularly, the software identifies the location and direction of minutiae (*e.g*., where a ridge ends or splits) and compares them between fingerprints^28^. The output is a similarity score (Fig. 2, bottom row of each section). The colored circles overlaying the binarized fingerprints represent minutiae identified by the system, vectors protruding from the circles identify the direction of minutiae, and the red box identifies the location(s) of the core origins^28,29^. Minutiae that match (based on direction and location relative to the core) between the original and phantom fingerprints are connected with lines to form a “tree”, differences in the distance between matched minutiae and the number of matches contribute to the similarity score^28,29^. The scores displayed in Fig. 2 are an average from three phantoms constructed independently of each source fingerprint. According to the specifications of Verifinger, a similarity score of 33 represents a false acceptance rate of 0.01%, which is considered sufficient for fingerprint matching. The score for all phantoms (>190) are well above this matching threshold, indicating the ridges are faithfully copied and detectable when imaged by a standard optical fingerprint scanner. Engelsma *et al*. also achieved similarity scores ranging from 100–300 when matching their 3D-printed phantoms and the original fingerprint image^10^. This comparison confirms that our naturally derived (*via* PC molding) PDMS phantoms should fulfill the purpose of testing the performance of fingerprint scanners and the embedded matching algorithms.

### Microscopic imaging of the mold and phantom

The exceptional quality of PDMS phantoms was further validated based on the reproduction (between mold and phantom) of the three levels of physical details that exist within a fingerprint pattern^1,30^. The first level of details is the location and arrangement of fingerprint ridges in relation to each other; the second level details (minutiae) are unique ridge patterns formed where ridges come together and differentiate^31^. Dimensional attributes of fingerprint ridges represent the third level of detail, including the width, edge contours, shape, the location and size of sweat pores and other permanent details such as creases or scars^30,31^. Highly distinctive third level features are excellent for examining partial prints; as few as 20–40 pores (size and location) are adequate for a positive identification^31^.

**Figure 3:**
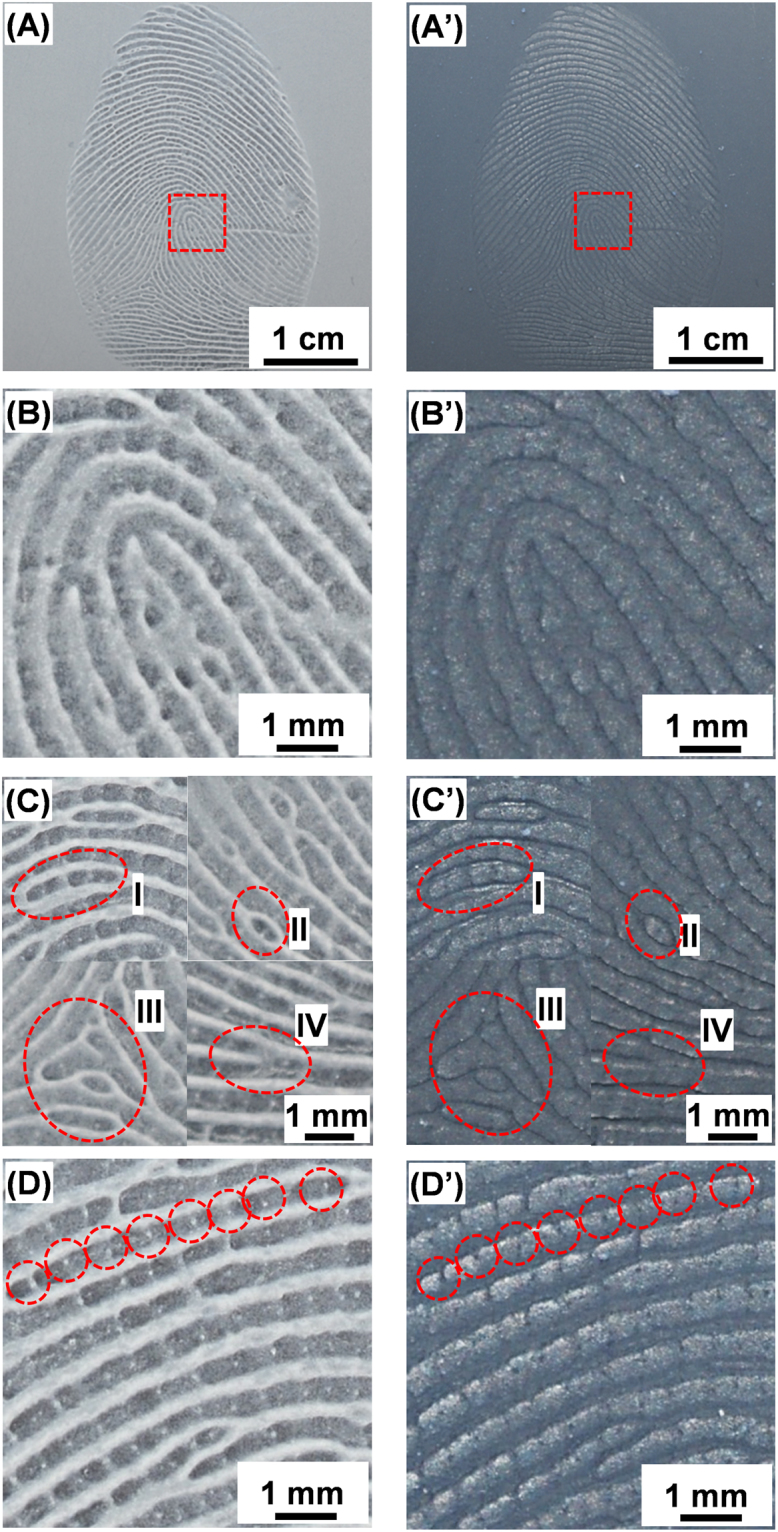
Optical imaging of the PC fingerprint mold (left) and PDMS phantom (right). (**A**)/(A’) shows the entire fingerprint, which represents the first level details; (**B**)/(B’) a magnified section showing the fingerprint core; (**C**)/(C’) selected second level details such as islands (I), incipient (under developed) ridges (II), delta (III), and bifurcation (IV); (**D**)/(D’) a high magnification image showing the third level details, *i.e*., sweat pores along a fingerprint ridge (as highlighted).

The complete fingerprint mold and phantom images are displayed in Fig. 3A,A’, respectively to show the reproduced first level details, *i.e*., an overview of the discernible individual ridges. Figure 3B/B’ highlights the fingerprint core in the PC mold and PDMS phantom, the essential feature to align fingerprints for matching^31^. In Fig. 3C,C’ several second level details are clearly defined, *i.e*., an island(I), an incipient ridge (II), a delta (III), and a bifurcation (IV) as highlighted in red dash-line circles. Comparison of the first level and second level details between the mold and phantom illustrates the accuracy of our molding approach; all minutiae recorded in the mold are present in the phantom and their relative locations are identical. The minimal size and depth of third level details such as sweat pores can make them difficult to identify and define; these distinctive features however, can be perfectly recorded in the PC mold and reproduced in the PDMS fingerprint phantoms (a series of pores are highlighted along a ridge in Fig. 3D/D’). Other third level details, such as ridge width and contours are also replicated well.

**Figure 4:**
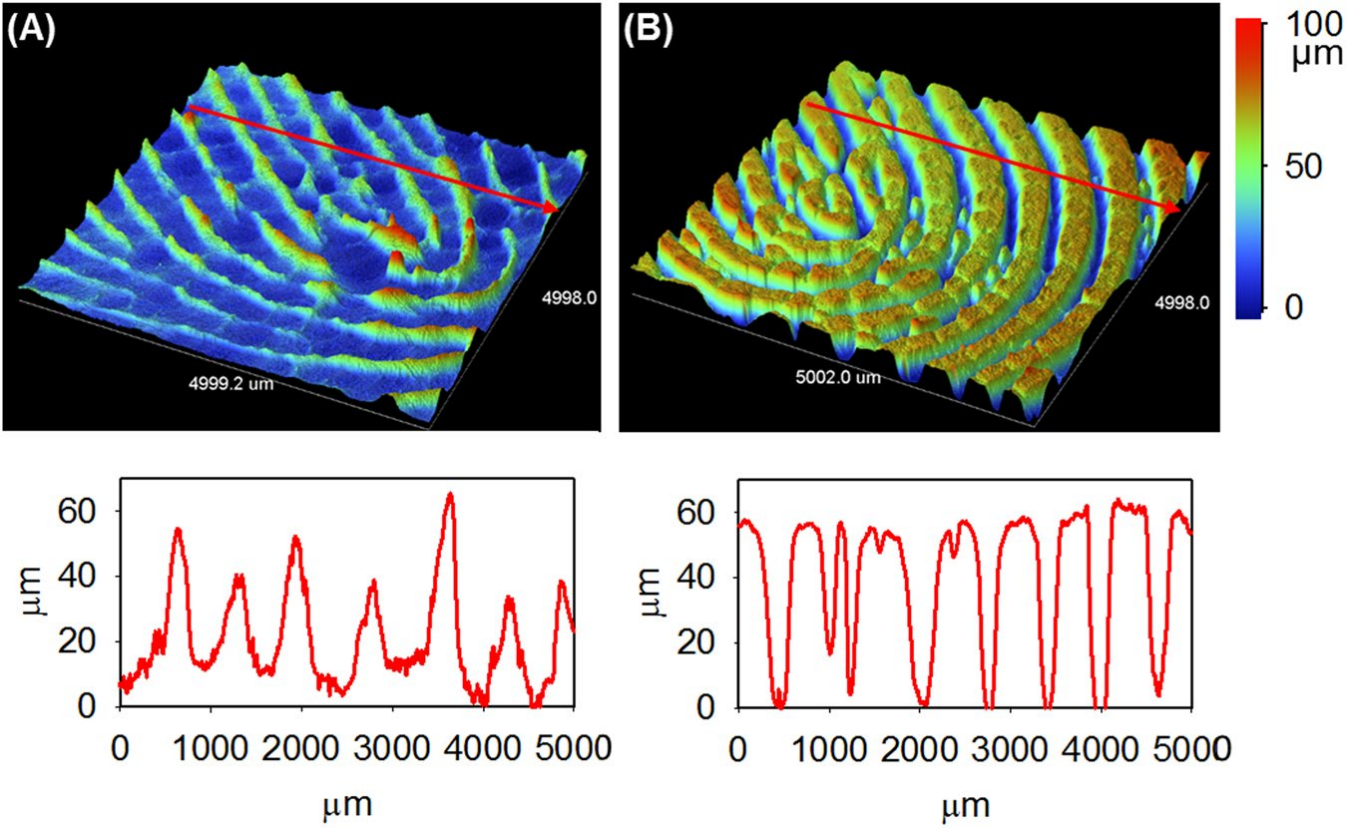
Profilometry imaging of PC fingerprint mold and PDMS phantom. Profiled 3D images of (**A**) PC mold, (**B**) PDMS phantom, and their respective cross section along the red line.

The other key feature of our phantoms is the capability of recording the depth of fingerprint ridges on the PC mold and reproducing them into PDMS phantom, i.e., the creation of a true three-dimensional (3D) polymeric replica of the original fingerprint. To illustrate this unique feature, both the fingerprint PC mold and PDMS phantom were imaged with a profilometer to examine the 3D morphology (Fig. 4). For the particular fingerprint replicated, the average ridge width is between 500 and 700 µm and the height ranges from 40 to 60 µm, which is well within the range of ridge dimensions of human fingerprints^32^. Along the ridges of the PC mold (Fig. 4A) minute changes in depth are visible, while the peaks (corresponding to the space between ridges in the original fingerprint) have more obvious variability in height (as shown in the inset below Fig. 4A). The PDMS phantom effectively copied the ridge impressions to reproduce subtle minutiae of the original ridges. The image in Fig. 4B shows that the ridges were reproduced down to the third level details of a fingerprint; differences in the steepness of ridges and the depth of valleys between them can be differentiated. Along ridges variations in height manifest as pore impressions and undulations in finger tissue. Subtleties such as the angle of ridge edges partially developed and shallow ridges were also well duplicated. The cross-section profile (inset below Fig. 4B) shows an incipient ridge and two pores located at 1200 µm, 1600 µm, and 2300 µm, highlighting the reproduced microscale details. 3D recording of third level details in fingerprint phantoms confirms that the topography of the PC mold (fingerprint impression) was effectively reproduced to the PDMS phantom. These PDMS phantoms have the advantage over other 2D image-derived phantoms because of their defined 3D morphological information^8,9^, which is imperative for confirming proper fingerprint reading in scanner development and subsequent testing.

The fingerprint PC molds and PDMS phantoms were also imaged with SEM to probe microstructural details and examine how accurately they were transferred during the molding and casting process. When swollen with a solvent (e.g., acetone), PC not only forms a malleable surface, but also undergoes rearrangement at the molecular level. Solvent molecules penetrate between polymer chains, which push them apart and increases their free volumes^25,26^. Greater free volumes allow PC which originally existed in an amorphous state (polymer chains are too rigid to crystallize from melting) to adopt ordered configurations and crystallize into spherulites (*i.e.*, spherical semi-crystalline regions inside non-branched linear polymers)^25,26^. Spherulites in PC range from 5 to 10 µm in size, consisting of ~100 nm crystalline PC tendrils which grow and branch outward from a central nucleation point with amorphous PC filling space between the tendrils^25,26^. Evidence of PC spherulite impressions in PDMS would indicate that PDMS can mold features at least as small as spherulites or their tendrils (at micrometer and nanometer scale, respectively).

**Figure 5:**
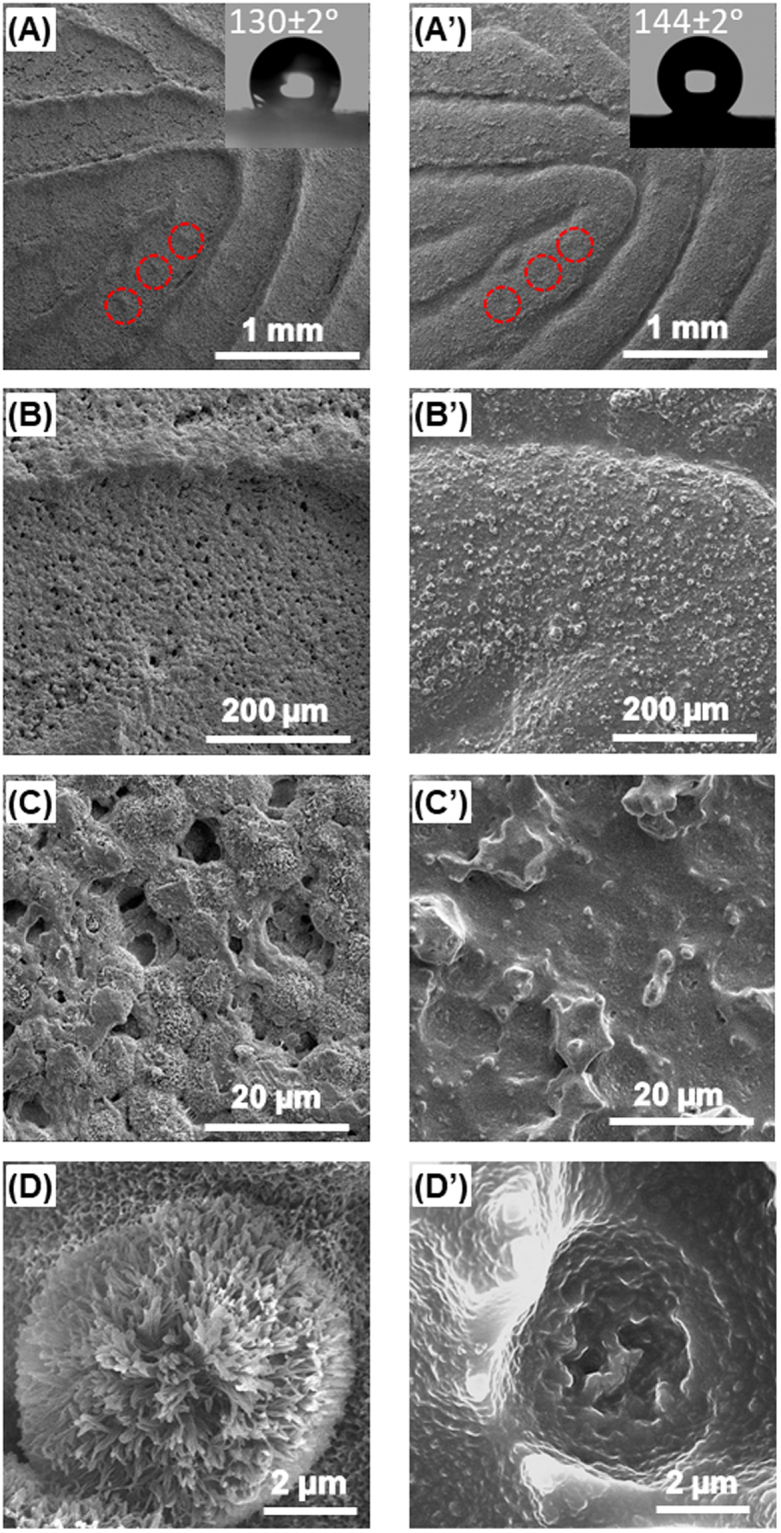
Scanning electron microscopy (SEM) imaging of PC fingerprint mold (left) and matching PDMS fingerprint phantom (right). (**A**)/(A’) Low magnification images of ridges; (**B**)/(B’) images of a single ridge surface; (**C**)/(C’) images of spherulites in PC mold and their impressions in the PDMS phantom; (**D**)/(D’) high magnification images of an individual spherulite and its impression. The insets in (**A**)/(A’) display water drops on the respective surfaces and corresponding contact angles.

Fig. 5A presents a low magnification SEM image of a fingerprint impression in PC; ridges are well defined; raised sections along ridges corresponding to sweat pores (in red circles) are visible. Fig. 5A’ displays the same area of the PDMS phantom casted from the PC mold in Fig. 5A. Visually phantom ridges match the PC impression and their similarity to actual ridges is striking; the sweat pores can be identified as slight depressions along the ridge (highlighted in red circles). Further examination of ridge impressions (Fig. 5B) revealed that PC forms a porous surface, as the PC chains rearranges into spherulites after swelling with acetone as noted above. The entire phantom surface is covered with uniformly distributed “protrusions” (Fig. 5B’), which conform to the porous surface of the PC mold. As shown in Fig. 5C, the PC spherulite surfaces are rough and they are interconnected with each other; their sizes vary from 5 to 10 µm. These high magnification SEM images show that the PDMS phantom (Fig. 5C’) in fact copies microscopic features as small as the spherulites from the PC perfectly. Even more remarkable, the shape of an individual spherulite can be “casted” on the PDMS phantom with the details corresponding to protruding tendrils (Fig. 5D/D’). The surface of spherulite impressions on the PDMS phantom (Fig. 5D’) are rough at the nanometer scale, as a result of PDMS conforming to the surface of spherulites (formed on the PC mold). These SEM studies illustrate that the PC molding procedure accurately reproduces the microscopic details of the original fingerprint.

The other surface property pertaining to the above described nanostructured morphology is the wettability. As shown as the inset in Fig. 5A, the water contact angle on the PC mold is 130 ± 2°, which is significantly higher than that of unmodified PC surface (90±2°). The PDMS phantom in fact has an even higher water contact angle (144 ± 2°, inset of Fig. 5A’). Such a near-superhydrophobic surface should be resistant to contamination, which ensures imaging reproducibility and renders long-term applications.

### Optimization of phantom fabrication and extended applications

As mentioned above, fingerprint scanners rely on various means of detecting ridges (optical property, conductivity, or ultrasound) and may use additional measures to verify the authenticity of fingerprints. Although PDMS is a great physical analog of skin in terms of strength and elasticity, their optical and electrical properties are very different, which can lead to unreadable phantoms. While the fingerprint matching shown in Fig. 2 was successfully performed with an optical scanner, we have identified possible additives to further improve their performance. To approximate tissue optical properties, a flesh colored silicone pigment (*e.g.*, pantone 488 C) can be added at low weight percent to the PDMS during curing (Fig. S1). Pigmentation provides the necessary light scattering and absorption characteristics for optical scanners to resolve ridges clearly^5,6^. Many different pigments can be chosen or combined to mimic a wide range of skin tones. Biological additives (blood cells, collagen, and lipids) cannot serve this purpose in PDMS phantoms as they do not disperse well and decompose rapidly^6^.

Fabrication of PDMS phantoms for reading with capacitive scanners is also feasible; these phantoms must achieve an electrical resistance of ~16 MΩ/cm to properly simulate human tissue^9^. There is a degree of flexibility in achieving readability by capacitive scanners because they are built to detect fingerprints with a high rate of success by accepting large variations in conductivity. Silver nanowires and particles may be applied, however large percentages (18%) are required to reach the percolation threshold and achieve the desired conductivity^33,34^. At such high additive ratios, PDMS becomes less elastic and conforms to the mold surface poorly^34^. Commercially available conductive PDMS precursors with silver coated aluminum nanoparticles dispersed at their percolation threshold may be tested^10^. Silicone thinner (5%) should be added to decrease viscosity and ensure complete molding of fingerprint impressions along with flesh colored pigment to produce an electrically and optically realistic fingerprint phantom^10^. Ultrasounic scanners should not require any additional modification as PDMS possesses a similar density to human tissue^11^.

The above described nanocontact replication protocol has applications beyond the creation of fingerprint phantoms; we believe that it can be expanded to be a bench-top fabrication technique for other pre-designed micro/nanostructures. Traditional micro/nanofabrication techniques require costly materials (e.g., high-grade silicon wafers, photoresists) and equipment (e.g., e-beam or UV lithography facilities), and previously reported microcontact molding techniques require well controlled molding parameters and conditions. For an example, most molding methods require a heated compression molding press to supply consistent heating and/or pressure^12–15^. Solvent-assisted molding techniques rely on well-defined solution conditions and polymer film thickness^12,22–24^. In contrast, our durable PC template can be fabricated from an unmodified sheet of PC under ambient laboratory conditions on benchtop upon brief treatment with a mild solvent. As an initial test, we have showcased the replication of a microstructured PC original to its one-to-one replica (see Supplementary information, Fig. S2).

Such a benchtop two-step replication protocol is conceptually different from the conventional soft lithography techniques (*e.g.*, microcotanct printing, PDMS replica molding, and direct solvent-assisted microcontact molding) for creating complementary patterns of the master as described in a seminal review by Qin *et al*.^13^. It should be pointed out that the potentials of bench-top fabrication techniques are unlimited. Most obviously, reducing the cost of producing nanostructures can help broaden the applications of micro/nanodevices (*e.g.*, optical filters, micro electromechanical systems, and microfluidics). Low-flow-resistance and low-fluid-loss microfluidic devices are immediate examples^35^, as microfluidics devices that perform protein separations rely on modified surfaces to localize and control surface adhesion^36^. PDMS “stamps” (templates) for micro-contact printing rely on embedded nanostructures to improve ink transfer and reduce residual contamination^37^.

## Conclusion

Unconventional PC molding has been shown to be an effective technique to construct 3D fingerprint phantoms, *i.e.*, fingerprint molds made of solvent-softened PC plates serve as robust templates to cast flexible and highly detailed PDMS replicas (fingerprint phantoms). By matching the PDMS phantoms with source fingerprints, we confirmed that the ridge patterns are faithfully reproduced and all three levels of fingerprint details are transferred in 3D. These refined phantoms are excellent tools to expedite and advance the development of biometric fingerprint scanners for security and law enforcement applications. This work also promises a novel benchtop nanocontact replication method that can be applied to the mass production of many other polymeric nanostructures and devices.

## Materials and Methods

Polycarbonate (PC) sheets were purchased from Bayer (Sheffield, USA), which are protected by plastic films on both sides upon receiving. Acetone (99.8%) and ethanol (95%) were purchased from Fisher Scientific. Sylgard 184 polydimethylsiloxane (PDMS) kit (which contains an elastomer base and a curing agent) was purchased from Ellsworth Adhesives (Germantown, USA). Deionized water (>18.3 MΩ·cm) was produced with a Barnstead Easypure UV/UF water system (Thermo Scientific, Waltham, USA).

To mold fingerprints a 1.5′′ × 1.5′′ piece of PC chip was cut from the large sheet and the protective film removed from one side. The bare surface was washed with deionized water and ethanol, then dried with N_2_. 1.0-mL of acetone was dispensed onto the PC surface with an automatic pipette and left for 45 s. A finger was then either pressed with mild force or rolled to produce a fingerprint impression on the surface. The PC plate was left to dry in ambient conditions to solidify the fingerprint impression (the mold). The fingerprint phantoms were constructed by casting a PDMS replica using the PC mold. Briefly, the two precursors supplied in the PDMS kit, the elastomer base (part A) and the curing agent (part B) were mixed in a 10:1 ratio by manual stirring and vortexing. The precursor solution was poured over the PC mold, degassed for 45 min in a vacuum chamber, and then cured in an oven at 80 °C for 2 h. The PDMS phantom is then cut and carefully peeled off from the PC mold.

Digital imaging of fingerprints and phantoms was conducted with a Hamster Plus Fingerprint Scanner (SecuGen Co., Santa Clara, USA). For each sample, a photo of the fingerprint/mold/phantom was captured and converted to an 8-bit gray-scale image at 500 DPI; the Verifinger 9.0 SDK software developed by NEURO technology (Vilnius, Lithuania) was used for fingerprint verification and matching.

Scanning electron microscopy was employed to examine the morphology of the PC molds and PDMS phantoms. An FEI NanoNova 430 SEM was used to obtain high resolution images; the samples were coated in a Hummer 6.2 gold sputtering system and sputtered in pulse mode for 6 min to deposit a conductive gold layer to the surface. The depth of the ridge impressions was measured using a Bruker Dektak XT (Billerica, USA) profilometer. Contact angle measurements were performed with a VCA Optima System (AST Products Inc.; Billerica, USA).

## Electronic supplementary material

Supplementary information
