# Supplementary material for: Fabrication of 3D Fingerprint Phantoms via Unconventional Polycarbonate Molding
Source: Sci Rep. 2018 Jun 25;8:9613. doi: 10.1038/s41598-018-27885-1 (PMC6018551; doi:10.1038/s41598-018-27885-1)
Supplement: Supplementary file 1 — Supplementary information [file 41598_2018_27885_MOESM1_ESM.pdf]

SUPPLEMENTARY INFORMATION

for

Fabrication of 3D Fingerprint Phantoms via Unconventional Polycarbonate Molding

Clayton W. Schultz, Jessica X. H. Wong, and Hua-Zhong Yu*

Department of Chemistry, Simon Fraser University, Burnaby, British Columbia V5A 1S6, Canada

* Corresponding author; e-mail: hogan_yu@sfu.ca

Additional experimental details including the fabrication of skin-color PDMS phantoms and an one-to-one replication of a PC micropattern. Further optical and spectroscopic characterization of the PC mold and PDMS replica are also presented.

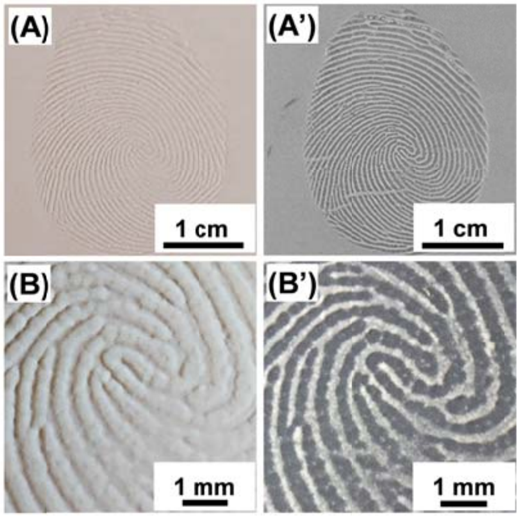

Figure S1. Photos of a skin-color PDMS phantom (left) and the PC mold (right). (A/A') shows the entire fingerprint, which represents the first level details; (B/B') a magnified section showing the fingerprint core. The pigment used to dope the phantom, Silc Pig Pantone 488C, is a commercial silicone dye for the entertainment industry to make props and fake limbs. The skin-color phantom in Figure S1(A) has similar optical properties as human skin. More importantly, the doping with pigments does not affect the casting of the phantom from the PC mold, i.e., the third level details (pores) are replicated accurately along the finger ridge impressions.

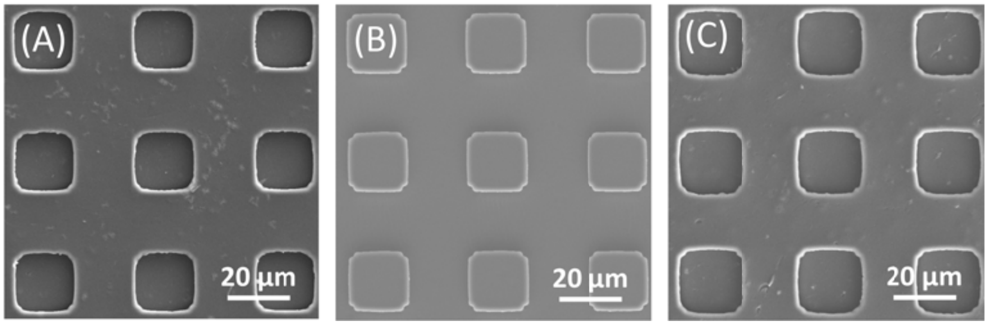

Figure S2. Replication of a designed micro-pattern. SEM images of (A) PC original (replicated from a PDMS template (prepared via microcontact molding of a silicon master that was made via standard lithography procedure); (B) PDMS mold; and (C) PC replica. It is evident that the micro-pattern on PC was molded to a PDMS template and then to a 1-to-1 PC replica with high fidelity.

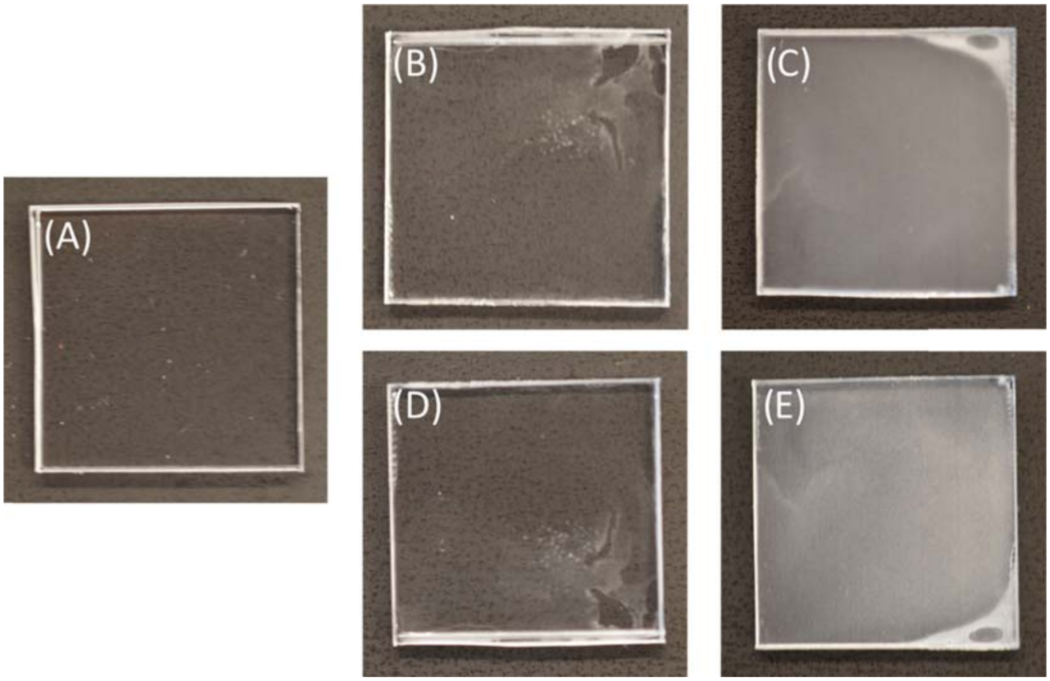

Figure S3. Photos of acetone-treated PC plates. PC plates were covered with acetone for a set period of time, then dried immediately in a stream of air to terminate the crystallization. (A) Untreated PC, (B) PC immersed in acetone for 15 s, (C) PC immersed in acetone for 45 s, (D) Underside of (B), (E) Underside of (C). The exposed side of the PC plate becomes opaque as the polymer chains begin to rearrange into semi-crystalline spherulites (B and C). Although we were not able to accurately determine the thickness of the crystalline layer based on these images, it is clear that crystallization occurs only at the surface as the back side is still clear (D and E). Preciously mechanistic studies indicate that acetone penetrates about ~100 µm into the bulk in 45 s (Turska *et al. J. Appl. Polym. Sci.* **1979**, *23*, 3489-3500), which is consistent with our observations.

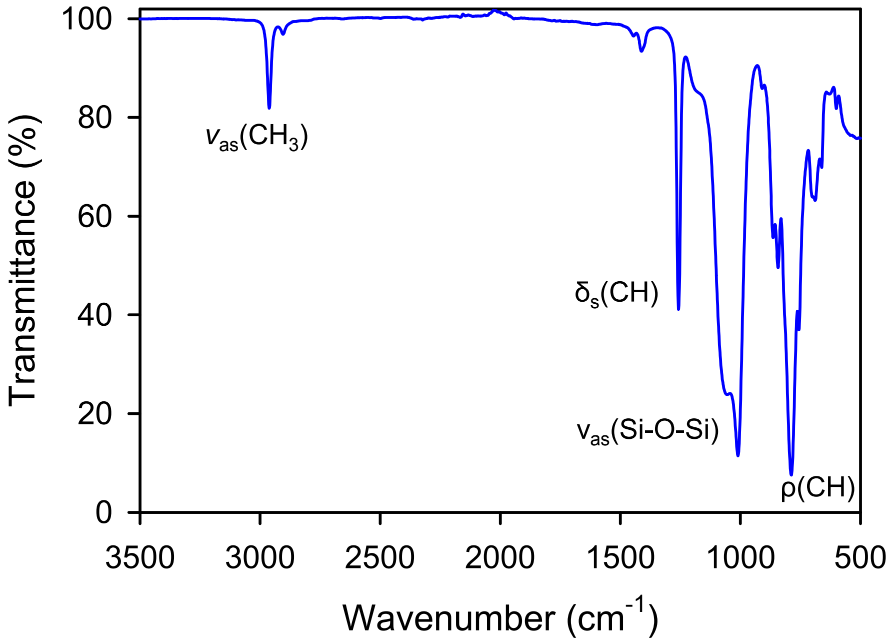

Figure S4. FT-IR spectrum of a PDMS plate casted from a PC mold. The major peaks associated with PDMS are present, *e.g.*, $v_{as}(CH_3)$ C-H bond stretch at 2950 cm^{-1}, δ_s(CH): bending of C-H at 1250 cm^{-1}, v_{as}(Si-O-Si): stretching vibrations of Si-O-Si bonds at 1000 cm^{-1}, and ρ(CH): C-H rocking at 800 cm^{-1} (Berdichevsky *et al.*, *Sens. Actuators B* **2004**, *97*, 402-408). No carbonyl peak is present (*v*(C=O) at 1670-1820 cm^{-1}) in the spectrum, indicating that no significant transfer of PC (or precursor) residues to the PDMS replica occurs during the casting process.
